# A probabilistic method for mapping earth fissure hazards

**DOI:** 10.1038/s41598-021-87995-1

**Published:** 2021-04-23

**Authors:** Mingdong Zang, Jianbing Peng, Nengxiong Xu, Zhijie Jia

**Affiliations:** 1 School of Engineering and Technology, China University of Geosciences (Beijing), Beijing, 100083 China; 2 School of Geological Engineering and Geomatics, Chang’an University, Xi’an, 710054 China; 3 Key Laboratory of Western China’s Mineral Resources and Geological Engineering, Ministry of Education, Xi’an, 710054 China

**Keywords:** Natural hazards, Geomorphology

## Abstract

Earth fissures caused by tectonic forces, human activities, or both seriously threaten the safety of people’s lives and properties. The Taiyuan Basin, a Cenozoic downfaulted basin located in the centre of the Fen-Wei Basin tectonic belt, in northwestern China, presents the ideal study area for a hazard assessment of earth fissures. A total of 104 earth fissures have been observed in the Taiyuan Basin, with a total length of approximately 128 km. In this paper, we proposed a probabilistic method for mapping earth fissure hazards by integrating the analytic hierarchy process (AHP), the area under the curve (AUC), and the certainty factor model (CFM). Geomorphic units, geologic formations, active faults and land subsidence zones of the Taiyuan Basin were mapped in detail. Correlations between these factors and earth fissures were evaluated through spatial modelling in ArcGIS. The AUC was introduced into the AHP to weight each factor and thus, to derive an earth fissure susceptibility map. Finally, the modelled earth fissure susceptibility was compared with a digital inventory of earth fissures to develop a probability function and map the spatial variability in failure probability through the CFM. The study indicates that active faults have the greatest contribution to the generation of earth fissures. Earth fissures are prone to develop in the piedmont alluvial-diluvial clinoplain and the transitional zone near the geomorphic boundary. This mapping procedure can assist in making rational decisions regarding urban planning and infrastructure development in areas susceptible to earth fissures.

## Introduction

Earth fissures are geotechnical surface ruptures formed as a result of internal and external geological forces. They have created serious hazards around the world, including in the United States^1–3^, China^4–12^, Mexico^13-15^, Ethiopia^16,17^, New Zealand^18^, Pakistan^19^, Saudi Arabia^20^, and Iran^21^. These earth fissures have caused significant damage to society, the economy, the environment, and humanity. In North China alone, earth fissures have destroyed large areas of farmland, water channels, buildings, and roads and have threatened the safe operation of the Metro and high-speed railway, resulting in direct economic losses of ¥10,000,000,000 (~ $1,447,073,200 USD) per year^22^. Earth fissures generally cluster and zonate in their distribution^22^. Estimating where earth fissures are most likely to develop plays an important role in the regional assessment of the hazards that they pose. This imperative problem has received international attention from both academia and engineering in recent decades^23,24^.

Several factors have been reported to be responsible for the origin of earth fissures: earthquakes^1,25,26^, fault activity^6,27,28^, pumping-induced land subsidence^29–32^, weak tensile characteristics of Quaternary sediment^23,33,34^, and paleogeomorphology^35,36^. It is thus challenging to accurately map the hazards posed by earth fissures due to the complexity, multidisciplinarity, and various uncertainties involved^37,38^. Wu et al.^39^ built a nonlinear modelling and forecasting system for earth fissures based on the coupling of artificial neural networks and GIS. Four factors (i.e., fault density, drawdown in groundwater level, stratum thickness, and geomorphic unit) were used to evaluate the earth fissure hazard potential, and five risk levels were identified in Yuci, Shanxi Province, China. Zhang et al.^40^ integrated artificial neural networks with genetic algorithms to evaluate the occurrence of earth fissures. The depth of bedrock burial, the degree of bedrock relief, water level, the gradient of land subsidence, transmissivity, and the thickness of clay soil were selected as indicators. Budhu^41^ developed a simple analytical model based on the Mohr–Coulomb failure criterion to understand the formation of earth fissures from groundwater level decline. The results have shown that the most efficient mechanism for the formation of earth fissures is combining bending with shearing; therefore, geological discontinuities are the preferred location for earth fissure formation. Peng et al.^6^ conducted a large-scale physical simulation experiment on the fracturing modes of earth fissures triggered by underlying fault activity in Xi’an. The experimental results indicated that the earth fissures were old fracture surfaces covered by a very thin layer of topsoil, and the over-pumping of groundwater has exposed them on the ground surface. Ye et al.^37^ introduced a novel numerical approach based on interface elements to simulate earth fissure generation and propagation in terms of both the sliding and opening of earth discontinuities. Their results highlight that bending of the alluvial deposits around the bedrock ridge tip, and shear stress due to uneven piezometric changes and asymmetrical bedrock shapes, are crucial for the generation and propagation of earth fissures. Choubin et al.^38^ proposed new machine learning models for the prediction of earth fissure hazards, as well as identifying important variables and the role of human activities. The results indicate that hazardous earth fissures are mainly related to areas with low elevation and with characteristics that include high groundwater withdrawal, high well density, high road density, low precipitation, and Quaternary sediment distribution.

In addition, the analytic hierarchy process (AHP) is also an effective method to map earth fissure hazards^42,43^. The generation and development of earth fissures are influenced by multiple factors, which creates a multicriteria problem. The AHP, originally proposed by Saaty^44^, is a classic and powerful method for this multicriteria analysis. The AHP has been widely used in the assessment of typhoon damage^45^, landslide hazards^46–48^, spontaneous coal combustion hazards^49^, snow avalanches^50^, flood hazards^51–53^, and other geo-environmental problems^54,55^. However, the conventional AHP has two main disadvantages: first, expert subjectivity in pairwise comparisons fails to quantify the weight of each factor^47,50^; second, without a completed database, the results from the AHP method are expressed as a range of scores, not the probability of hazard occurrence.

The present work introduces a probabilistic method for mapping earth fissures hazards based on the integration of the AHP, the area under the receiver operating characteristic (ROC) curve, known as the AUC, and the certainty factor model (CFM). The ROC curve is a function of the sensitivity and specificity for each value of a variable, and the AUC provides a single quantitative index of the performance of the variable^56–58^. Therefore, the AUC can be employed to quantitively weigh the factors in terms of earth fissures, which can overcome the subjectivity uncertainties of conventional AHP. This method has been applied by different researchers in landslide hazard mapping^59–62^. Furthermore, the CFM, created by Shortliffe and Buchanan^63^ and improved by Hecherman^64^, is one of the possible proposed favourability functions to handle the problem of combining different data layers, as well as the heterogeneity and uncertainty of the input data^65^. The CFM is coupled with a digital inventory of earth fissures developed in the Taiyuan Basin to transfer susceptibility values from the AHP into earth fissure spatial probability and thus, to overcome the second drawback of conventional AHP. We hope that our method can be useful in assessing earth fissure hazards in similar regions with scarce data and provides guidelines for decisions regarding infrastructure siting and long-term land use planning.

## Study area

**Figure 1:**
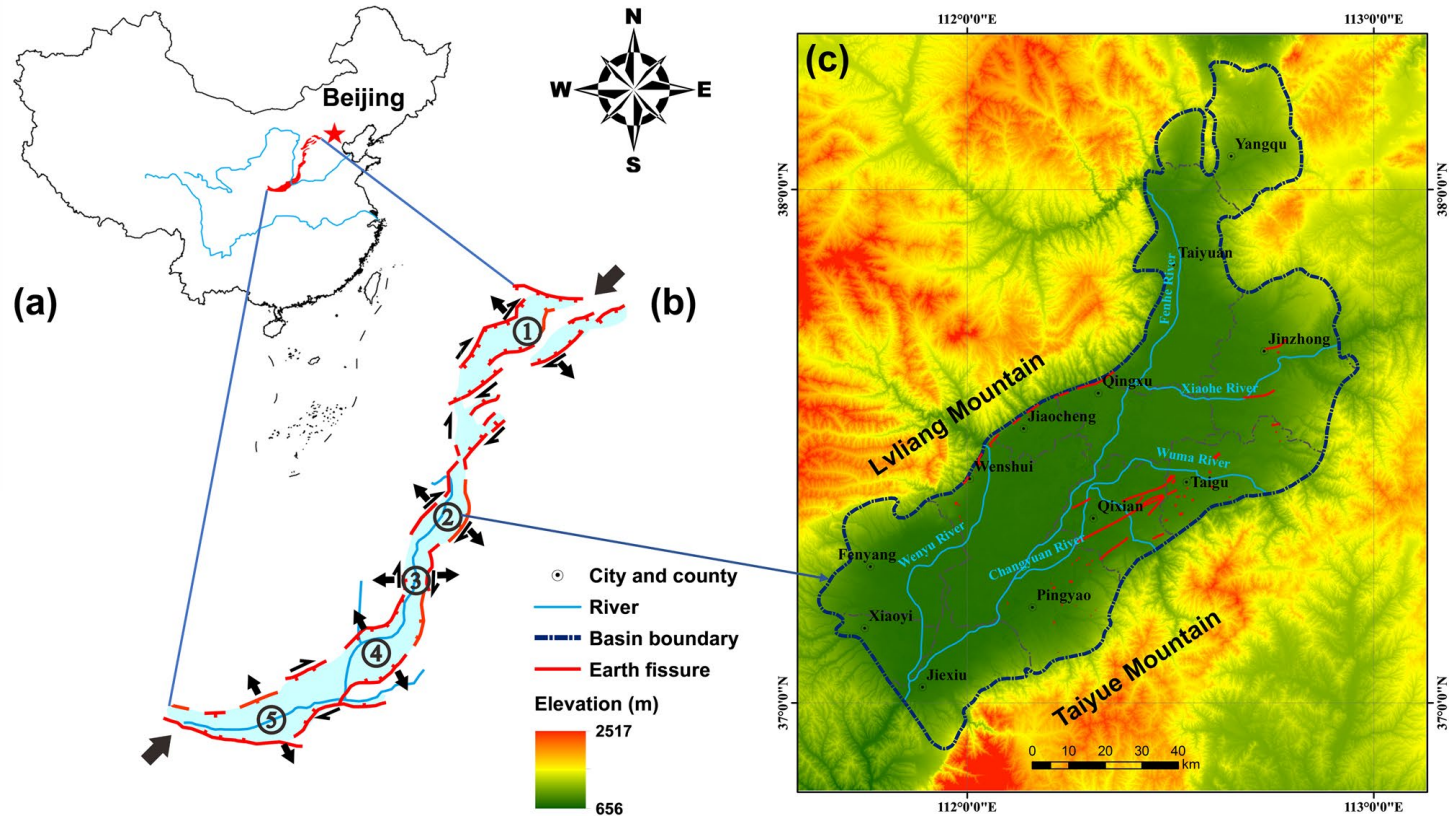
Distribution of earth fissures in the study area.

The Taiyuan Basin is a famous Cenozoic fault basin located in the middle of Shanxi Province, northern China (Fig. 1a). It is approximately 148 km long in the northeast (NE)–southwest (SW) direction and approximately 42 km wide in the northwest (NW)–southeast (SE) direction, covering a total area of 6200 km^2^. The basin is bounded by Lvliang Mountain to the west and Taiyue Mountain to the east (Fig. 1c). Datong Basin is located to the north of the Taiyuan Basin and Linfen Basin, Yuncheng Basin, and Weihe Basin are located to the south (Fig. 1b). These five basins together are referred to as the Fen-Wei Graben System, which is a typical Cenozoic fault-basin system in Asia^7^. The Taiyuan Basin experiences tension in the NW–SE direction, which is mainly controlled by the counter clockwise rotation of the Ordos Block, induced by the uplift of the Tibetan Plateau, and the uncoordinated movements of the North China Block and the South China Block in a southeastern direction^66^ (Fig. 1b).

**Figure 2:**
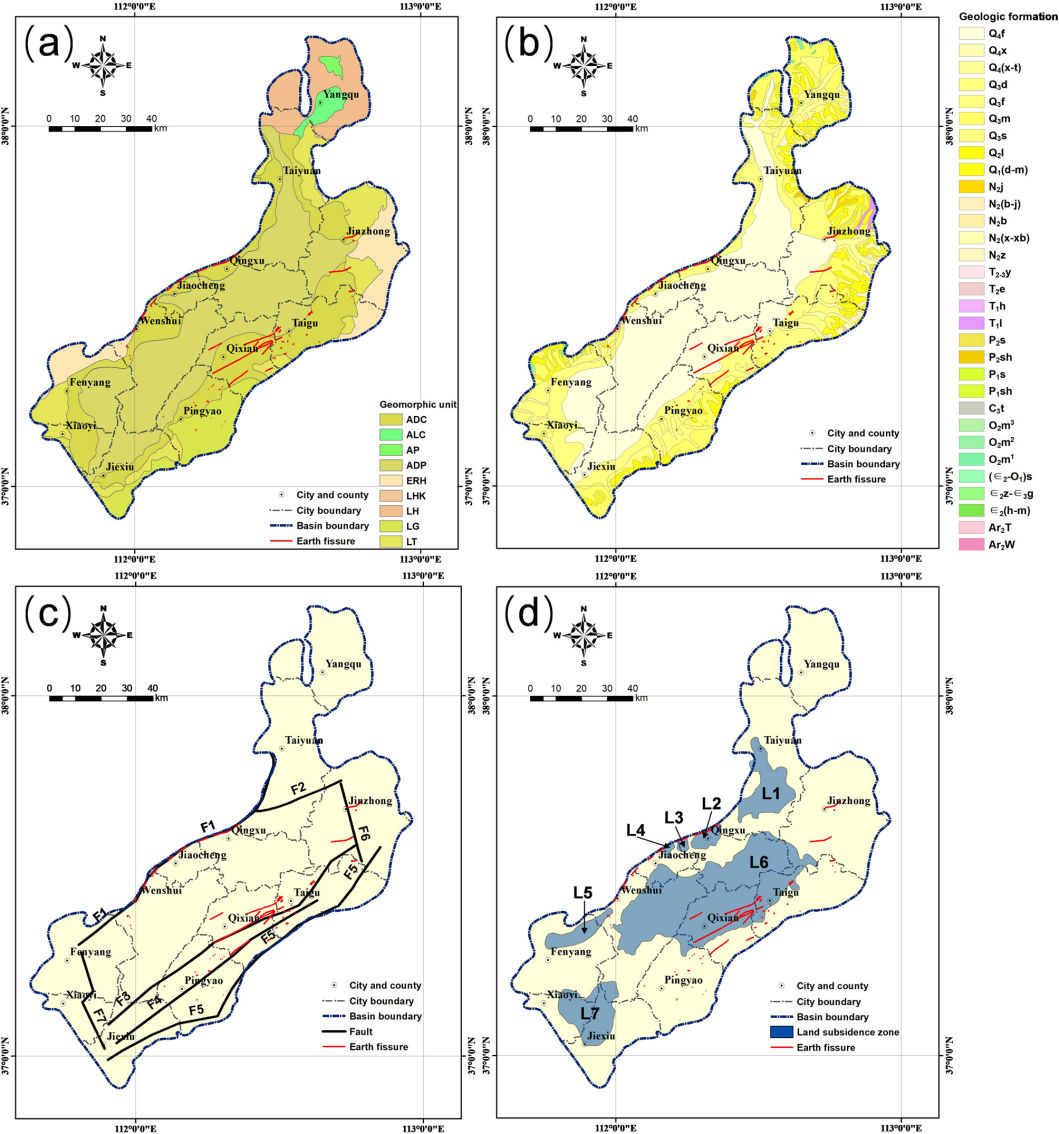
Maps showing (**a**) geomorphic units, (**b**) geologic formations, (**c**) faults, and (**d**) land subsidence zones in the study area. The geomorphic, fault, and land subsidence zone maps are adapted from the Geological Environment Monitoring Center of Shanxi Province, China. The geologic map is adapted from the China Geological Survey.

The terrain of the Taiyuan Basin generally decreases in elevation from north to south while increasing in elevation from the centre to the periphery. The elevation gradually declines from the mountains to the interior of the basin, with an average of 825 m (Fig. 1c). The highest elevation is 2517 m in the Guandi Mountain area, which is the middle part of Lvliang Mountain, and the lowest elevation is 656 m in the Jiexiu area south of the basin. The Fenhe River flows into the Yellow River from north to south, with its tributaries located throughout the basin, resulting in large-area alluvial-diluvial plains (Figs. 1c and 2a). According to field investigations, landforms in the study area can be subdivided into nine types (Fig. 2a): the piedmont alluvial-diluvial clinoplain (ADC), alluvial-lacustrine clinoplain (ALC), alluvial plain (AP), alluvial-diluvial plain (ADP), earth-rock hill (ERH), loess hillock (LHK), loess hill (LH), loess gully (LG), and loess tableland (LT). The central basin is mainly covered by ADPs, adjoining ADCs on their sides. Clinoplains border the mountain area in the Qingxu-Jiaocheng-Wenshui and Taigu-Qixian-Pingyao areas. LHKs, LGs, and LTs develop between the mountain area and clinoplains with varied widths in other areas (Fig. 2a).

Extra-thick Cenozoic deposits were formed during the prolonged subsidence of the Taiyuan Basin, with an average thickness of 1000–2000 m and a maximum thickness of 3800 m in the Jiaocheng depression, northwest of the basin^67^. Quaternary sediments are widely distributed over the central basin (Fig. 2b), with a thickness of approximately 400 m^68^. As listed in Table 1, the central basin is covered by the Dagou-Mugua Formation of early Pleistocene age, the Lishi Formation of middle Pleistocene age, the Malan, Shiyu, and Fangcun formations of late Pleistocene age, and the Dingcun, Xuanren, Xuanren-Tuoyang, and Fenhe formations of Holocene age. Mountain areas at the margin of the Taiyuan Basin have sparse distributions of Triassic, Permian, Carboniferous, Ordovician, Cambrian and Archaeozoic strata (Fig. 2b).

**Table 1:** Geologic formations in the study area (adapted from the China Geological Survey).

Symbol	Formation	Description
${Q}_{4}f$	Fenhe Formation	Sandy gravel, sand, sandy loam, and clay loam
${Q}_{4}x$	Xuanren Formation	Sandy gravel, sand, sandy loam, and clay loam
${Q}_{4}(x-t)$	Xuanren-Tuoyang Formation	Sandy gravel, sand, sandy loam, and clay loam
${Q}_{3}d$	Dingcun Formation	Clay loam with sandy gravel
${Q}_{3}f$	Fangcun Formation	Sandy gravel, sandy loam, and clay loam
${Q}_{3}m$	Malan Formation	Loess and sandy loam
${Q}_{3}s$	Shiyu Formation	Sandy gravel, silty sand with clay loam, clay, and loess-like soil
${Q}_{2}l$	Lishi Formation	Loess, sandy loam, clay loam with sandy gravel, paleosol belt, and calcareous nodules
${Q}_{1}(d-m)$	Dagou-Mugua Formation	Fine sand with gravels and thin layer of clay
${N}_{2}j$	Jingle Formation	Sandy clay and clay with calcareous nodules
${N}_{2}(b-j)$	Baode-Jingle Formation	Merging layer of Baode and Jingle formations
${N}_{2}b$	Baode Formation	Sandy gravel, sandy clay, clay, and silty loam with calcareous nodules
${N}_{2}(x-xb)$	Xiatuhe-Xiaobai Formation	Clay, silty loam with fine sand and sandy gravel
${N}_{2}z$	Zhangcun Formation	Clay with marl, sand, and sandy clay
${T}_{2-3}y$	Yanchang Formation	Arkose with mudstone, and thin coalbeds
${T}_{2}e$	Ermaying Formation	Arkose with sandy mudstone, and massive calcareous nodules
${T}_{1}h$	Heshanggou Formation	Mudstone, and sandy mudstone with arkose
${T}_{1}l$	Liujiagou Formation	Arkose with silty mudstone
${P}_{2}s$	Sunjiagou Formation	Mudstone, and silty mudstone with arkose
${P}_{2}sh$	Shihezi (upper) Formation	Shale with sandstone
${P}_{1}s$	Shanxi Formation	Sandstone, shale, and coalbeds
${P}_{1}sh$	Shihezi (lower) Formation	Quartz sandstone, feldspathic quartz sandstone with sandy shale, and mud shale
${C}_{3}t$	Taiyuan Formation	Sandstone, shale, carbonaceous shale with coalbed, and limestone
${O}_{2}{m}^{3}$	Majiagou (upper) Formation	Thick layer of limestone with leopard limestone
${O}_{2}{m}^{2}$	Majiagou (middle) Formation	Muddy dolomite, argillaceous limestone, and dolomitic limestone
${O}_{2}{m}^{1}$	Majiagou (lower) Formation	Brecciated marl, limestone, and dolomite
${(\in }_{2}-{O}_{1})s$	Sanshanzi Formation	Dolomite
${\in }_{2}z-{\in }_{3}g$	Zhangxia-Gushan Formation	Oolitic limestone, wormkalk, and argillaceous limestone
${\in }_{2}(h-m)$	Huoshan-Mantou Formation	Sandstone and shale
${Ar}_{2}T$	Taiyueshan Formation	Tonalitic and granodioritic gneiss
${Ar}_{2}W$	Wenyuhe Formation	Gneiss and granite

According to geophysical prospecting conducted by the Geological Environment Monitoring Center of Shanxi Province, China, seven active Quaternary faults, F1, F2, F3, F4, F5, F6 and F7, are present in the Taiyuan Basin. From Fig. 2c, the faults can be generally grouped into two categories based on their direction of strikes: one category that strikes in a NE to northeast-east (NEE) direction that included Faults F1, F2, F3, F4, and F5; and another category that strikes in a NW direction that consists of Faults F6 and F7. Fault F1, which is a large normal fault that controls the northwestern boundary of the Taiyuan Basin^69^, originates from Shanglan, Taiyuan, crosses through Qingxu, Jiaocheng, and Wenshui, and ends at Fenyang with a length of 125 km, generally striking 55° and dipping to the SE with an angle of over 80°. According to its geometry and activity, Fault F1 can be divided into four sections^70^, i.e., the Jinci, Qingxu-Jiaocheng, Wenshui, and Fenyang sections, from north to south. The active age and activity rate of each section are described in detail in the next section. Fault F2, which is a buried normal fault, originates from Tiancun, Taiyuan, and ends at Tianzhuang with a length of approximately 35 km, generally striking NEE and dipping to the SE at an angle of 50–80°. Fault F3 is a buried normal fault with a length of approximately 110 km, generally striking 54° and dipping to the SE at an angle of 50–60°. Fault F4 is also a buried normal fault with a length of approximately 100 km, generally striking 50° and dipping to the NW at an angle of approximately 70°. Fault F5, which is a normal fault that controls the southeastern boundary of the Taiyuan Basin^68^, originates from Taigu and ends at Lingshi with a length of approximately 130 km, generally striking 45° and dipping to the NW at an angle of 70–82°. According to its geometry and activity, Fault F5 can be divided into three sections^71^, i.e., the Fancun, Taigu, and Hongshan-Dongquan sections, from north to south. The active age and activity rate of each section are discussed in detail in the next section. In the second category-faults striking in a NW direction-Fault F6 is a buried normal fault with a length of approximately 34 km, striking north-northwest (NNW) and dipping to the SW, and Fault F7 is a normal fault, generally striking NNW and dipping to the NE with an angle of over 80°.

According to surface deformation monitoring data from 2009 to 2013 (Geological Environment Monitoring Center of Shanxi Province, China), there are seven subsidence zones-the Taiyuan land subsidence zone (L1), Qingxu land subsidence zone (L2), Qingxu-Jiaocheng land subsidence zone (L3), Jiaocheng land subsidence zone (L4), Fenyang land subsidence zone (L5), Qixian-Taigu land subsidence zone (L6), and Jiexiu-Xiaoyi land subsidence zone (L7) that are interpreted to exist in the Taiyuan Basin (Fig. 2d). Among these subsidence zones, L6 has the largest area, covering 1847 km^2^, while L2 has the highest annual subsidence rate, with an average of 7 cm/a.

## Results

The generation of earth fissures is mainly influenced by geomorphic units, geologic formations, faults, and land subsidence^6,10,11,23,36,72,73,74^. The dataset and mapping procedure are detailed in the Methods. The results are described as below.

### Normalization of the units in each factor

**Figure 3:**
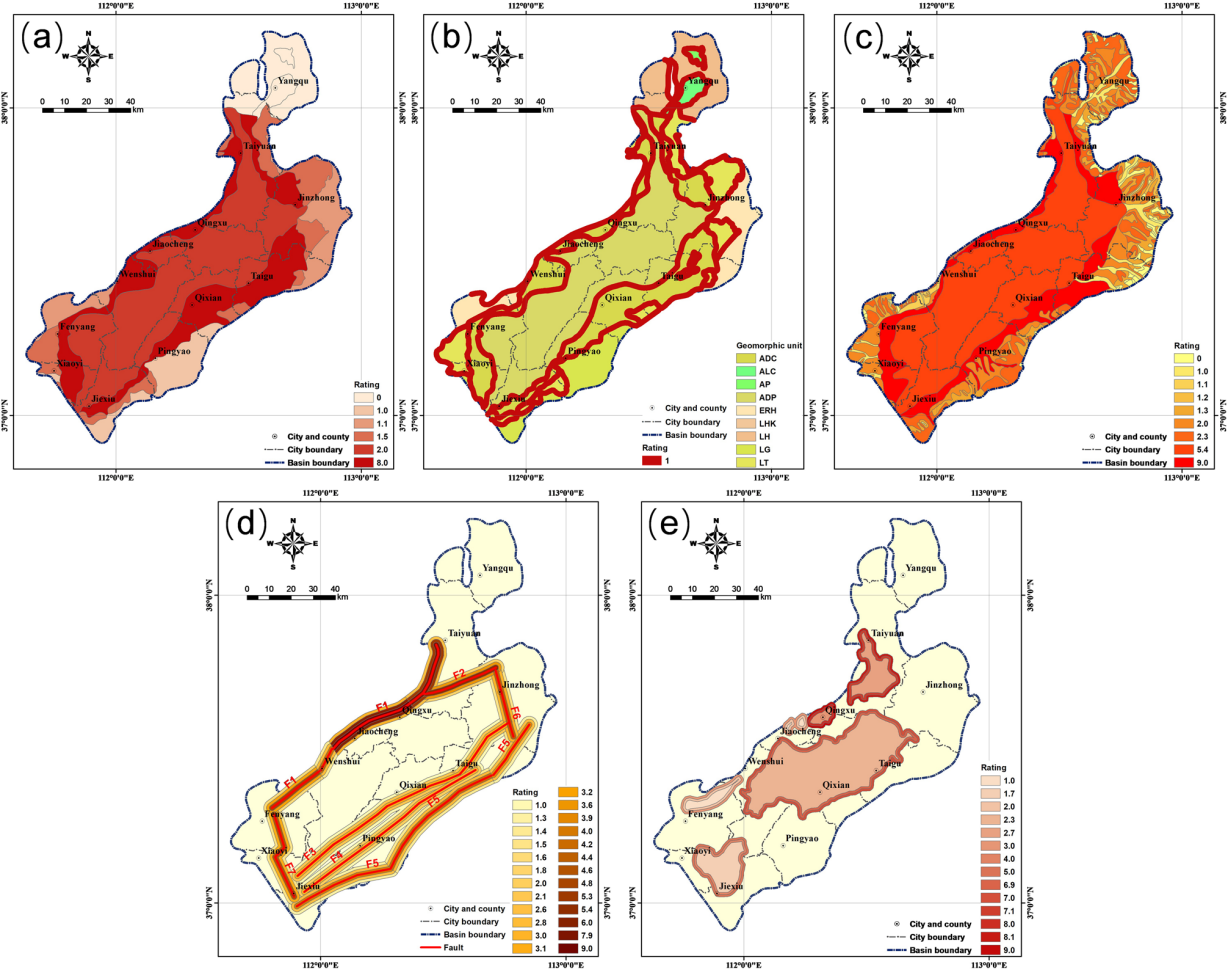
Maps showing ratings of (**a**) geomorphic units, (**b**) transitional zones, (**c**) geologic formations, (**d**) faults, and (**e**) land subsidence zones in the study area.

The units in each factor were normalized by a number from 1 to 9 according to their contribution to the generation of earth fissures. Geomorphic units were normalized based on the proportion of the cumulative length of earth fissures to the total length of earth fissures. The maximum proportion occurred in the ADC, reaching 78%; therefore, the ADC was rated as 8. The minimum nonzero proportion occurred in the LG with a value of 1.2%, which was rated as 1. Other geomorphic units were rated between 1 and 8, as listed in Table 2. No earth fissure was found in the AP, LH, LHK and ALC; therefore, these units were rated as zero. In addition, the transitional zones around the geomorphic boundaries were rated as an additional rating of 1. The ratings of the geomorphic units are shown in Fig. 3a,b.

**Table 2:** Ratings of geomorphic units.

Symbol	Geomorphic unit	Length of earth fissures (m)	Percentage of total fissure length (%)	Rating
ADC	Piedmont alluvial-diluvial clinoplain	99,777.4	78.0	8.0
ADP	Alluvial-diluvial plain	15,242.1	11.9	2.0
LT	Loess tableland	8812.7	6.9	1.5
ERH	Earth-rock hill	2491.5	1.9	1.1
LG	Loess gully	1586.4	1.2	1.0
–	Transitional zone	53,610.1	41.9	Additional 1.0
–	Others	0	0	0

Geologic formations were also normalized based on the proportion of the cumulative length of earth fissures to the total length of earth fissures. The proportion of the length of earth fissures within the Fangcun Formation was as high as 51%, which was the maximum proportion among the formations, while the minimum nonzero proportion occurred in the Xuanren-Tuoyang Formation, where the length of earth fissures was 0.2 m. Therefore, the Fangcun Formation was rated as 9, and the corresponding rating of the Xuanren-Tuoyang Formation was 1. Other geologic formations were rated between 1 and 9, as listed in Table 3. It should be noted that twenty-one geologic formations that are scattered at the basin margins (e.g., the Yangchang, Ermaying, and Heshangou formations) with no earth fissures were rated as zero. The ratings for each geologic formation are presented in Fig. 3c.

**Table 3:** Ratings of geologic formations.

Symbol	Unit name	Length of earth fissures (m)	Percentage of total fissure length (%)	Rating
${Q}_{3}f$	Fangcun Formation	65,217.2	51.0	9.0
${Q}_{4}f$	Fenhe Formation	35,555.6	27.8	5.4
${Q}_{3}m$	Malan Formation	10,893.7	8.58	2.3
${Q}_{3}s$	Shiyu Formation	7888.8	6.2	2.0
${P}_{1}s$	Shanxi Formation	2466.4	1.9	1.3
${Q}_{2}l$	Lishi Formation	2106.7	1.6	1.3
${P}_{1}sh$	Shihezi (Lower) Formation	1967.6	1.5	1.2
${T}_{1}l$	Liujiagou Formation	1327.6	1.0	1.2
${Q}_{3}d$	Dingcun Formation	486.3	0.4	1.1
${Q}_{4}(x-t$)	Xuanren-Tuoyang Formation	0.2	< 10^–4^	1.0
–	Others	0	0	0

Faults were normalized based on the distance to the fault and the activity rate. The Qingxu-Jiaocheng section of Fault F1 had the highest activity rate, reaching 1.3 mm/a, and the latest active age was the middle Holocene (${Q}_{4}^{2}$). Consequently, Zone I of Fault F1 was rated as 9, Zone II was rated as 6, and Zone III was rated as 3, as illustrated in Table 4. Fault F4 had the lowest activity rate with a value of 0.03–0.07 mm/a, and the average value of 0.05 mm/a was used for calculating the rating. Compared with Fault F1, Zone I of Fault F4 was rated as 3, Zone II was rated as 2, and Zone III was rated as 1, as illustrated in Table 4. The ratings of other faults are presented in Table 4 and Fig. 3d.

**Table 4:** Ratings of faults (the active age and activity rate of the faults are from Wang et al.^68^).

Symbol	Fault		Active age	Activity rate (mm/a)	Rating		
					I	II	III
F1	Jiaocheng Fault	Jinci section	${Q}_{3}^{3}$	1.07	7.9	5.3	2.6
		Qingxu-Jiaocheng section	${Q}_{4}^{2}$	1.3	9.0	6.0	3.0
		Wenshui section	${Q}_{2}$	0.29	4.2	2.8	1.4
		Fenyang section	${Q}_{2}$	0.43	4.8	3.2	1.6
F2	Tianzhuang Fault		${Q}_{2}^{3}$	0.55	5.4	3.6	1.8
F3	Qixian-Dongyang Fault		${Q}_{2}$	0.07	3.1	2.1	1.0
F4	Pingyao-Taigu Fault		${Q}_{2}$	0.05	3.0	2.0	1.0
F5	Hongshan-Fancun Fault	Fancun section	${Q}_{2}$	0.24	3.9	2.6	1.3
		Taigu section	${Q}_{2}$	0.31	4.2	2.8	1.4
		Hongshan-Dongquan section	${Q}_{2}$	0.25	4.0	2.6	1.3
F6	Yuci-Beitian Fault		${Q}_{3}$	0.35	4.4	3.0	1.5
F7	Sanquan Fault		${Q}_{2}$	0.38	4.6	3.1	1.5

The land subsidence zone was normalized based on the position within the land subsidence zone and the subsidence rate. According to the monitoring data of surface deformation conducted by the Geological Environment Monitoring Center of Shanxi Province, China, among the seven subsidence zones of the Taiyuan Basin, L2 has the highest average annual subsidence rate, with a value of 7 cm/a. The outer peripheral zone of L2 was rated as 9, the inner peripheral zone was rated as 8, and the central zone was rated as 2.7. The average annual subsidence rates of subsidence zones L3, L4, and L5 are all within 2.0–3.0 cm/a, so an average value of 2.5 cm/a was used for calculation. The outer peripheral zones of these three subsidence zones were rated as 3, inner peripheral zones were rated as 2, and central zones were rated as 1. L6 presents an average annual subsidence rate of 5.0–6.0 cm/a, and an average value of 5.5 cm/a was taken for calculation. According to the linear interpolation, the outer peripheral zone of L6 was rated as 7, the inner peripheral zone was rated as 6, and the central zone was rated as 2.3. The ratings of other land subsidence zones are illustrated in Table 5 and Fig. 3e.

**Table 5:** Ratings of land subsidence zones (the average subsidence rate of the land subsidence zones are from the Geological Environment Monitoring Center of Shanxi Province, China).

Symbol	Land subsidence zone	Average subsidence rate (cm/a)	Rating		
			Outer peripheral zone	Inner peripheral zone	Central zone
L1	Taiyuan	6.3	8.1	7.1	2.7
L2	Qingxu	7.0	9.0	8.0	3.0
L6	Qixian-Taigu	5.5	7.0	6.0	2.3
L7	Jiexiu-Xiaoyi	4.0	5.0	4.0	1.7
L3, L4, L5	Others	2.5	3.0	2.0	1.0

### Susceptibility of earth fissures

**Figure 4:**
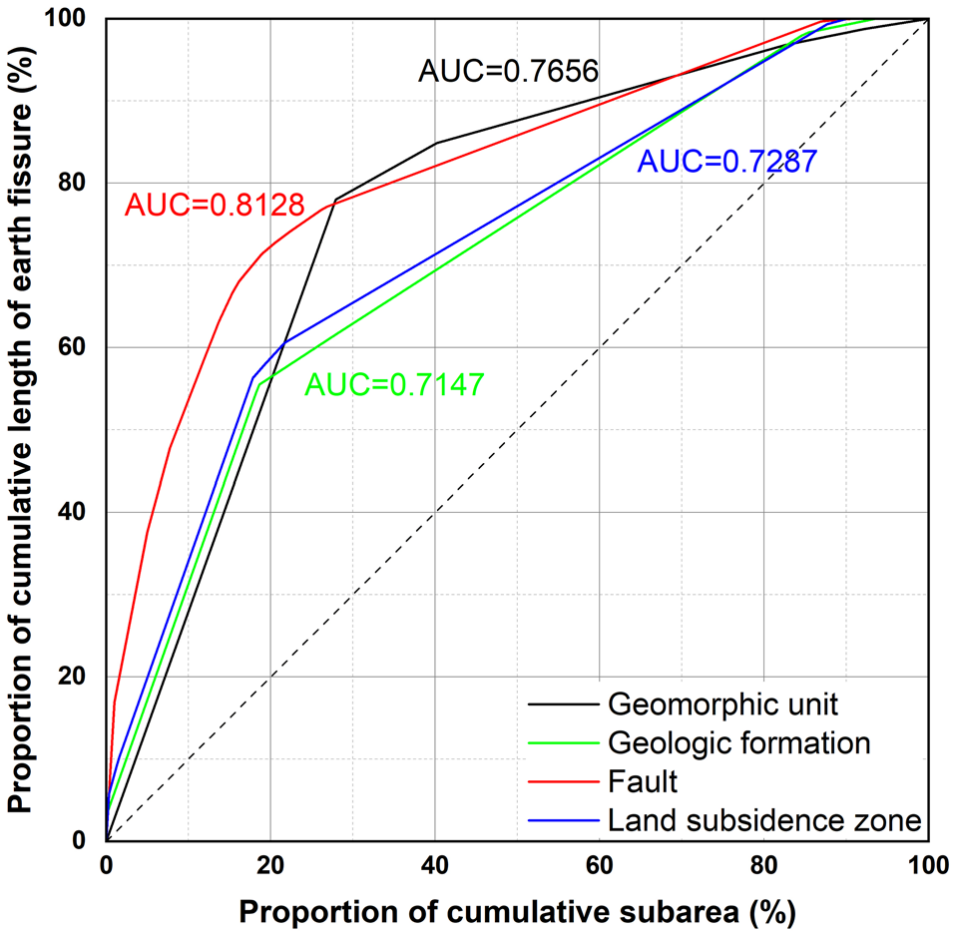
Area under the curve plots for comparing the factors.

**Figure 5:**
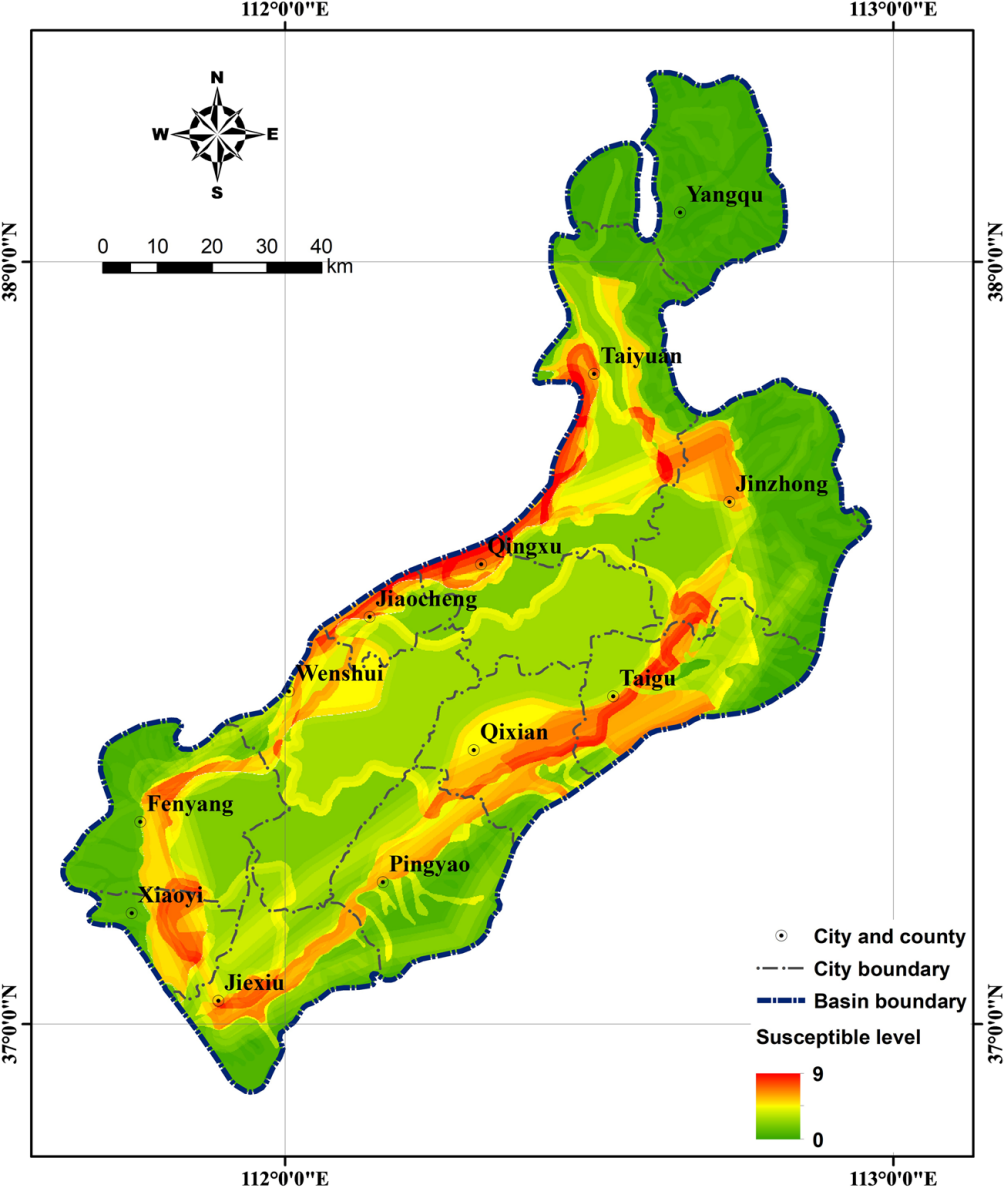
Map showing earth fissure susceptibility in the study area.

The AHP was integrated with the AUC to predict the susceptibility of earth fissures. From Fig. 4, the AUC values of the geomorphic unit, geologic formation, fault, and land subsidence zone are 0.7656, 0.7147, 0.8128, and 0.7287, respectively. The AUC value was calculated as an index to represent the relative dominance of a factor. A factor with a higher AUC value indicated a more dominant role in the susceptibility of earth fissures. A pairwise comparison matrix was built based on the AUC ratio. The scale of weight was derived through the normalized principal eigenvector of the matrix. As listed in Table 6, the relative weights of the geomorphic unit, geologic formation, fault, and land subsidence zone are 0.2534, 0.2365, 0.269, and 0.2411, respectively. Reasonability of the reciprocal pairwise comparison matrix was measured by the consistency ratio ($CR$) proposed by Saaty^74^. For the proposed reciprocal matrix, the maximum eigenvalue of the matrix was ${\lambda }_{max}$ = 4, the number of factors was $n$ = 4, and thus, the consistency index was $CI$ = 0 and, in turn, $CR$ = 0. Therefore, the reciprocal pairwise comparison matrix was consistent, and the weights for the factors were reasonable. Afterwards, the susceptibility values of the earth fissures were calculated, with a range from 0 to 9. Larger values indicated a higher susceptibility of earth fissures. A map showing earth fissure susceptibility was then generated, as shown in Fig. 5.

**Table 6:** Reciprocal pairwise comparison matrix of the factors.

AUC	Factor ${\varvec{i}}/{\varvec{j}}$	Geomorphic unit	Geologic formation	Fault	Land subsidence zone	Weight
0.7656	Geomorphic unit	1	1.0712	0.9419	1.0506	0.2534
0.7147	Geologic formation	0.9335	1	0.8793	0.9808	0.2365
0.8128	Fault	1.0617	1.1373	1	1.1154	0.2690
0.7287	Land subsidence zone	0.9518	1.0196	0.8965	1	0.2411

### Probability of earth fissures

The CFM was coupled with a modified Weibull^75^ curve to establish a probability function of earth fissures. The certainty factor ($CF$) represents the confidence level for earth fissures. The calculated certainty factors ranged from − 1 to 0.97. Positive values corresponded to an increase in the confidence level for earth fissures, while negative quantities corresponded to a decrease in this confidence. Higher positive values indicated higher confidence levels for earth fissures.

**Figure 6:**
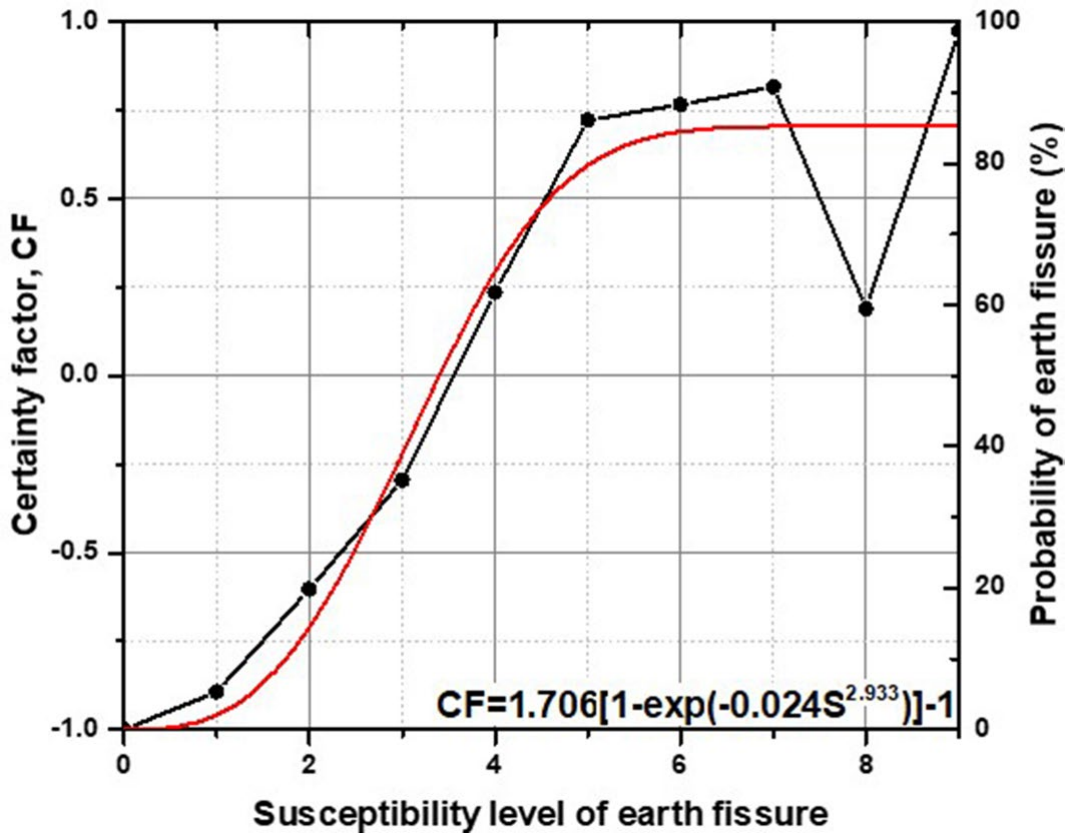
$CF$ as a function of the level of susceptibility.

**Figure 7:**
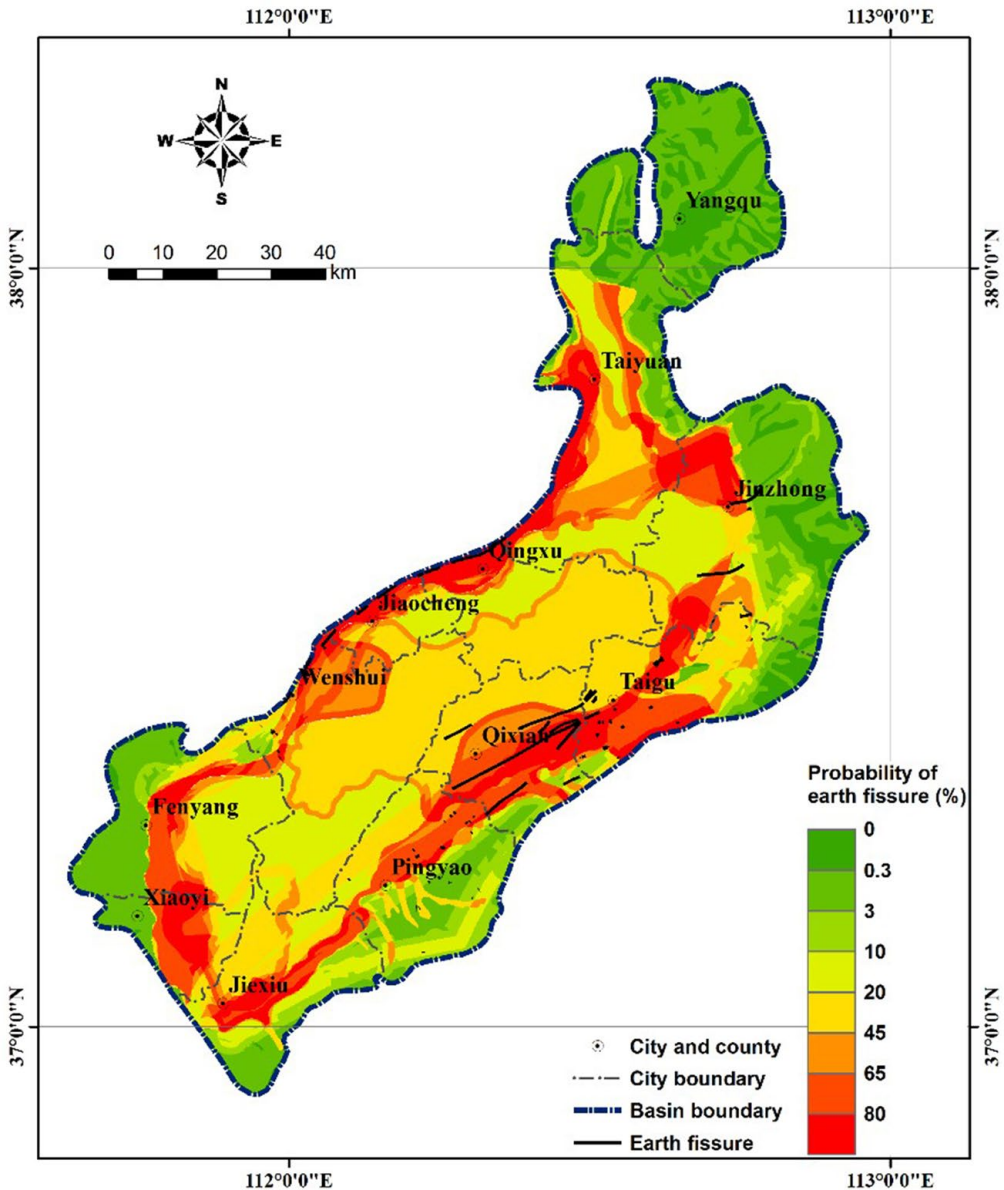
Map showing probability of earth fissures in the study area.

Figure 6 shows, in each bin, the $CF$ plotted as a function of susceptibility. A Weibull^75^ curve modified by Zang et al.^76^ was chosen to fit the data in Fig. 6. The regression curve based on data from the Taiyuan Basin is

1 $$CF=1.706[1-\exp(-0.024{S}^{2.933})]-1$$

where $CF$ is the certainty factor and $S$ is the level of earth fissure susceptibility. The curve fits the data well (${R}^{2}$ = 90%), and the probability corresponding to $CF$ is shown on the y-axis on the right side in Fig. 6. Equation (1) can be used to estimate the probability of earth fissures as a function of the level of susceptibility and provides the basis for producing an earth fissure hazard map. Figure 7 shows a map of the earth fissure hazards in the Taiyuan Basin. As shown in Fig. 7, the values of $CF$ range from − 1 to 0.7, with a corresponding failure probability ranging from 0 to 85%. High-hazard zones with a higher probability of earth fissures are mainly distributed along the margin of the basin as bandings. Medium-hazard zones are relatively concentrated in the central part of the basin. Low-hazard zones are located in the northern, northeastern and southwestern parts of the basin.

## Discussion

For geomorphic units, the maximum proportion of the cumulative length of earth fissures appeared in the ADC, as much as 78%, and the transitional zone came in second (Table 2). The ADC is an alluvial-diluvial fan located on the slope of a foothill, developing between mountains and the ADP. This loose and unconsolidated sediment provides favourable conditions for earth fissures. Additionally, layers with different physical and mechanical properties have alternately been deposited in the transitional zone, coupled with large surface reliefs, causing fragile geological conditions that predispose them to earth fissures.

For geologic formations, the maximum proportion of earth fissures was observed in the Fangcun Formation, and the Fenhe Formation came in second (Table 3). As listed in Table 3, the cumulative length of earth fissures developed in these two units accounted for 78.8% of the total length of earth fissures. By comparing Fig. 2b with Fig. 2a, we see that the Fangcun and Fenhe formations were primarily deposited in the ADC and ADP, respectively. The spatial distribution pattern of earth fissures was similar within geomorphic units and geologic formations. This indicates that the lithology of the strata plays an indispensable role in the generation of earth fissures.

For faults developed in the Taiyuan Basin, the Jinci section and Qingxu-Jiaocheng section of Fault F1 both had high activity rates, with an activity rate exceeding 1 mm/a (Table 4). The vast earth fissures that developed on the northwestern boundary area of the Taiyuan Basin were approximately parallel to Fault F1 (Fig. 2c). Although the activity rates of Faults F3, F4 and F5 were not as high as Fault F1, they developed close to each other, breaking the surface and favouring the formation of earth fissures. Therefore, the earth fissures developed in this local area are closely related to fault activity.

For land subsidence zones, the Qingxu zone had the highest average subsidence rate, and the Taiyuan zone came in second (Table 5). From Fig. 2d, we see that a large group of earth fissures crossed through Zones L2, L3 and L4, and another large group of earth fissures developed in Zone L6. The majority of earth fissures in these zones were found along the margins of the zones. Zone L1 also has a high average subsidence rate, close to that of Zone L2, and higher than that of Zone L6. However, no earth fissure has been found in this zone until now. We suspect this is because the soil layer here is relatively flat; therefore, it easily forms horizontal tension in the soil but not vertical dislocation. Horizontal deformation alone is unlikely to result in earth fissures.

The ROC curve indicates how well a factor interprets the data^77^. A higher AUC value indicates a higher success rate^61^. In the evaluation of a specific factor, Lee et al.^77^ classified AUC > 0.8 as excellent, 0.8 > AUC > 0.7 as good, 0.7 > AUC > 0.6 as fair, 0.6 > AUC > 0.55 as poor, and AUC < 0.55 as very poor. As listed in Table 6, the AUC values of the four factors selected in this study are all greater than 0.7, which indicates that they are effective in the construction of a susceptibility model. As the AUC values were calculated from the ratings of the units, it can be inferred that the ratings listed in Tables 2, 3, 4, 5 are reasonable. In addition, faults were found to be most closely correlated with earth fissures among the factors, with AUC values as high as 0.8128. The Taiyuan Basin is a Cenozoic fault basin cut by the northwestern margin fault Jiaocheng Fault, the southeastern margin fault Hongshan-Fancun Fault and five other active faults, forming a rhombus area and limiting the distribution range of the earth fissures. Figure 7 shows the probabilities of earth fissures in the Taiyuan Basin. By comparing Fig. 7 with Fig. 2c, we see that the pattern of higher probability (red coloured) areas is similar to that of the fault distribution, which also indicates the dominant function of local tectonic structures.

The inventory of earth fissures was covered on the map to demonstrate their goodness of fit for the predicted probability of earth fissures (Fig. 7). The earth fissures with a cumulative length of 93.7 km, which accounts for 73% of the total length, lies in areas with a probability value greater than 60%. Earth fissures with a cumulative length of 5.7 km, which accounts for 4% alone of the total earth fissure length alone lies in areas with a probability value less than 10%. These earth fissures that lie in the area with relatively low probability were mainly distributed at the basin margins. They were far away from faults and land subsidence zones, and their direction of strikes were unfixed. These earth fissures are usually controlled by a single factor or a couple of factors, especially the geomorphic unit or the geologic formation.

A regression curve relating the probability of earth fissures and the level of susceptibility was drawn based on data from the Taiyuan Basin (Fig. 6). The probability equation [Eq. (1)] can be applied to predict the earth fissure hazards in other scenarios of interest. However, recalibration for use in a different region is desirable, as regression constants may differ from those in another region if the geological setting varies significantly from that in the study area. In addition to some important discoveries revealed by this study, there are also limitations. The feature types of geomorphic units, geologic formations and land subsidence zones are polygons, while those of faults are polylines. As the feature type for earth fissures is also a polyline, the same as for faults, this may predispose the mapping procedure to be fault-driven. This predisposition deserves attention when using the method in other regions.

GIS-based multicriteria analysis is a powerful approach for the spatial assessment of geohazards that are influenced by multiple factors. The AHP is the most classical multicriteria analysis method. In the AHP method, pairwise comparisons form the backbone of the methodology^78,79^. However, the main disadvantage of the technique is the uncertainties raised by the expert subjectivity in pairwise comparisons. Grey relational analysis and the fuzzy logic approach have been introduced to construct the pairwise comparison matrix and to derive the relative weights of factors^45,46,50^. The statistical index method has been used to calculate the weighting value for the classes of each factor^47^. However, these integrations mostly involve the standardization of relevant conditioning factors or compensation of the vagueness problem due to the boundaries of the factor scores^50^. In addition, factors are generally combined by applying a weight to each with a weighted linear combination to yield a single score of evaluation^46,52,54,79^. However, the score is hardly combined with the hazard occurrence. The proposed method in this study is also a GIS-based multicriteria analysis approach, concentrating on the quantitative evaluation of the probability of hazard occurrences in a region. Compared with an inventory of earth fissures, a correlation analysis allowed us to determine the scales for the units of each factor. The AUC was integrated into the conventional AHP to quantify the weight of each factor instead of subjective judgements. A map of earth fissure susceptibility was produced based on a weighted linear combination of each factor. In addition, we used the CFM to develop a probability function to map earth fissure hazards. The proposed approach incorporates rating and weighting and becomes quantitative with the integration of a hazard inventory. Maps produced using this method can be useful in urban planning, infrastructure development, and a variety of other applications.

## Conclusion

Based on the analysis above, it can be concluded that faults have dominant contribution to the formation of earth fissures. The piedmont alluvial-diluvial clinoplain and the transitional zone of geomorphic units are prone to earth fissures. We used an integrated analysis of the AHP and the AUC to generate a quantitative scale of the relative dominance of factors. The CFM was introduced to develop a probability equation for earth fissures with the help of an inventory of earth fissures, thus producing a hazard map of earth fissures. The integration of AHP, AUC and CFM makes it possible to complete probabilistic hazard mapping of earth fissures. This mapping procedure has practical applications in the prediction of regional hazards caused by earth fissures.

## Methods

### Dataset

Datasets needed to conduct hazard mapping of the earth fissures include (1) a comprehensive inventory of earth fissures, (2) a digital geomorphic map, (3) a digital geologic map, (4) a digital map showing the distribution of faults, and (5) a digital map showing land subsidence zones by observed land surface elevations and their changes. All of these datasets are rasterized at 30 m grid spacing using ArcGIS software.

### Normalization of the units in each factor

The layer for each factor contains different units. For these units, the contribution to the generation of earth fissures was normalized by a number from 1 to 9 through a correlation analysis. Details are described as follows.

Two aspects were considered when rating the contribution from the geomorphic units. First, the proportion of the length of the earth fissures within each geomorphic unit to that of the total earth fissures was taken as an indicator. The maximum proportion was rated as 8 and the minimum nonzero proportion was rated as 1. Other geomorphic units were rated between 1 and 8 based on their proportions of the length of earth fissures using linear interpolation. Units without earth fissure were rated as zero. Second, sediment with a significant variation in thickness and surfaces with a large relief-sometimes scarps-are often found near the geomorphic boundary, which largely favours the development of earth fissures^7,22^. Therefore, the area within 500 m on either side of a geomorphic boundary was demarcated as a transitional zone with an additional rating of 1. The contribution from transitional zones was added to that of the geomorphic units in subsequent spatial analysis.

The proportion of the length of earth fissures within each geologic formation to that of the total earth fissures was used as the index for rating. The maximum proportion was rated as 9 and the minimum nonzero proportion was rated as 1. Ratings of other geologic formations were calculated through linear interpolation between 1 and 9 according to the proportion of the length of earth fissures. The geologic formations with no earth fissures were rated as zero.

Two main factors were considered when rating the contribution from each fault: the distance to the fault and activity rate of the fault. First, research has found that the influence of fault movement on the generation and development of earth fissures decreases with increasing distance from the fault^22^. According to this influence, the area within 1 km of either side of a fault was demarcated as the primary affected zone, named Zone I; similarly, the area 1–2 km away from a fault was demarcated as the secondary affected zone, named Zone II; and the area 2–3 km away from a fault was demarcated as the tertiary affected zone, named Zone III. Moreover, the activity rates of the faults and their sections were surveyed. Zone I of the fault, which had the highest activity rate, was rated as 9, Zone II was rated as 6 (two-thirds of the rating of Zone I), and Zone III was rated as 3 (one-third of the rating of Zone I). Zone I of the fault, which had the lowest activity rate, was rated as 3, Zone II was rated as 2 (two-thirds of the rating of Zone I), and Zone III was rated as 1 (one-third the rating of Zone I). Zone I of the other faults was rated by linear interpolation between 3 and 9 according to the activity rate. Zone II was given two-thirds of the rating of Zone I, and Zone III was given one-third of the rating of Zone I.

According to Wu et al.^43^, large deformation often occurs in the margin of a subsiding basin, where failure develops in the soil when the tensile stress, induced by differential settlement, exceeds the tensile strength of the soil. The developmental degree of earth fissures varies with the location within a subsiding basin. The specific position within a land subsidence zone and the subsidence rate are two important factors that need to be considered when rating the contribution from subsidence zones. The circumferential area within 1 km outside the boundary of a land subsidence zone was specified as the outer peripheral zone; the area within 1 km inward from the boundary was specified as the inner peripheral zone; and the rest of the area of the land subsidence zone was specified as the central zone. The rating of the outer peripheral zone should be higher than that of the inner peripheral zone, and the rating of the inner peripheral zone should be higher than that of the central zone. Furthermore, land subsidence zones with a higher settling rate should have a higher rating. The outer peripheral zone of the subsidence zone, which has the highest average annual subsidence rate, was rated as 9, the inner peripheral zone was given a rating of 1 less than that of the outer peripheral zone, i.e., 8, and the central zone was rated with one-third of the rating of the outer peripheral zone, i.e., 2.7. The outer peripheral zones of the subsidence zones, which has the lowest average annual subsidence rate, was rated as 3, inner peripheral zones were rated as 2 (the rating of the outer peripheral zone minus 1), and central zones were rated as 1 (one-third of the rating of the outer peripheral zone). Outer peripheral zones of other subsidence zones were rated by linear interpolation between 3 and 9 with respect to their subsidence rates. The rating of the inner peripheral zone was that of the outer peripheral zone minus 1 and that of the central zone was one-third of that of the outer peripheral zone.

### Weighing the factors

The AHP was employed to weigh the factors and then to compute the earth fissure susceptibility in the study area. Conventional AHP consists of four steps^80^: (1) pairwise comparison with a fundamental scale value from 1 to 9; (2) generating a reciprocal pairwise comparison matrix; (3) deriving the scale of weights; and (4) checking the consistency of the reciprocal pairwise comparison matrix.

Pairwise comparisons are fundamental in the use of the AHP^74^. Factors, i.e., geomorphic units, geologic formations, faults and land subsidence zones, were compared in pairs for their relative importance, thus generating a reciprocal pairwise comparison matrix. For a conventional AHP, comparisons are expressed verbally as equal, moderate, strong, very strong, and extreme, with a scale of number that indicates how many times more dominant one factor is over another^74^. Here, the AUC was employed to give a quantitative scale for each factor to construct a pairwise comparison matrix.

To create an AUC plot, the cumulative area of ratings within each calculated value from the maximum to the minimum was determined as a proportion of the total rating area (x-axis) and plotted against the proportion of cumulative length of earth fissures falling within those ratings (y-axis), as shown in Fig. 4.

In Table 6, the factors shown on the left were, one by one, compared with each factor shown on the top, and the AUC ratio was then used to conduct a pairwise comparison matrix. Comparing geomorphic units and geologic formations, the AUC of the geomorphic units was divided by that of the geologic formations; thus, we set 1.0712 in the geomorphic unit and geologic formation position and the reciprocal value 0.9335 in the geologic formation and geomorphic unit position. In this way, a reciprocal pairwise comparison matrix was built by entering the entire number in its appropriate position and automatically entering its reciprocal in the transposed position^81^.

After making the reciprocal pairwise comparison matrix, we derived the scale of weights, which is obtained by calculating the normalized principal eigenvector of the matrix. The normalized principal eigenvector showed relative weights among the factors.

Reasonability of the results was measured by the consistency ratio ($CR$) proposed by Saaty^74^. The $CR$ was obtained by comparing the consistency index ($CI$) with the random consistency index ($RI$) to see if it was approximately 10% or less^74^. The $CI$ of a reciprocal matrix can be computed as follows^74^:

2 $$CI=\frac{{\lambda }_{max}-n}{n-1}$$

where $CI$ is the consistency index, ${\lambda }_{max}$ is the maximum eigenvalue of the matrix, and $n$ is the number of factors. $RI$ can be derived from a sample size of 500 of a randomly generated reciprocal matrix using scales 1/9, 1/8, …, 1 ,…, 8, 9, as shown in Table 7.

**Table 7:** Random consistency index^74^.

*n*	1	2	3	4	5	6	7	8	9	10
*RI*	0	0	0.58	0.90	1.12	1.24	1.32	1.41	1.45	1.49

The susceptibility value for each grid cell in the study area was calculated by summing the products of the ratings of the components within each factor and the weight of the factor. A map showing earth fissure susceptibility was then generated, as shown in Fig. 5.

### Estimating the probability for earth fissures

To produce an earth fissure hazard map, we chose the CFM^63,64^ to explore the relationship between the occurrences of earth fissures and their susceptibility values. The CFM was created for managing uncertainty in a rule-based system^63^. In this model, the certainty factor $CF$ represents the net confidence in hypothesis $H$ based on evidence $E$^64^. Certainty factors range between − 1 and 1. A $CF$ with a value of − 1 represents a total lack of confidence, whereas a $CF$ with a value of 1 represents total confidence. Values greater than zero favour the hypothesis, while those less than zero favour its negation. The probabilistic interpretation of $CF$ is as follows^64^:

3 $$CF=\left\{\begin{array}{c}\frac{p\left(H|E\right)-p\left(H\right)}{p\left(H|E\right)\left[1-p\left(H\right)\right]}, \quad p\left(H|E\right)>p\left(H\right)\\ \\ \frac{p\left(H|E\right)-p\left(H\right)}{p\left(H\right)\left[1-p\left(H|E\right)\right]}, \quad p\left(H|E\right)<p\left(H\right)\end{array}\right.$$

where $CF$ is the certainty factor, $p\left(H|E\right)$ is the posterior probability that relies on evidence, and $p\left(H\right)$ is the prior probability before any evidence is known. In the probability analysis of earth fissures, $p\left(H|E\right)$ was defined as the linear density of earth fissures within a specific susceptibility value area, and $p\left(H\right)$ was defined as the linear density of earth fissures within the entire study area. They can be calculated by dividing the area of a specific susceptibility value area or the entire study area by the total length of earth fissures in this area. In this way, the values of $CF$ represented the confidence level for earth fissures. Following this definition, susceptibility cells at every level were grouped into bins. For each bin, the linear density of earth fissures was calculated, which was considered the posterior probability of the bin as defined. The prior probability calculated by dividing the entire length of the earth fissures by the entire study area was the same in each bin.

According to Zang et al.^76^, a regression function of $CF$ and the level of susceptibility would make it possible to predict the spatial probability in earth fissures in any given scenario of interest. We chose a Weibull^75^ curve modified by Zang et al.^76^ to fit the data in Fig. 6. The functional form is

4 $$CF=2m[1-\exp(-a{S}^{b})]-1$$

where $CF$ is the certainty factor, $m$ is the maximum $CF$ value represented by the data, $S$ is the level of earth fissure susceptibility, and $a$ and $b$ are regression constants.

According to the definition of $CF$, a value of − 1 represents a total lack of confidence; therefore, the corresponding probability was set as 0, whereas a value of 1 represents total confidence; therefore, the corresponding probability was set as 100%. Other corresponding probabilities of $CF$s were obtained through linear interpolation. In this way, this regression curve of $CF$ was applied to generate a map showing the probability of earth fissure hazards.
